# Inpatient TIA and stroke care in adult patients in Germany - retrospective analysis of nationwide administrative data sets of 2011 to 2017

**DOI:** 10.1186/s42466-019-0044-y

**Published:** 2019-12-01

**Authors:** Jens Eyding, Dirk Bartig, Ralph Weber, Aristeidis H. Katsanos, Christian Weimar, Werner Hacke, Christos Krogias

**Affiliations:** 1 Department of Neurology, Klinikum Dortmund gGmbH and Universityhospital Knappschaftskrankenhaus Bochum, Beurhausstr, 40, D-44137 Dortmund, Germany; 2 Northwest-German Stroke Circle e.V, Bochum, Germany; 3 drg market, Osnabrück, Germany; 4 Department of Neurology, Alfried Krupp Hospital Essen, Ruhr University Bochum, Essen, Germany; 5 Department of Neurology, University Hospital St. Josef-Hospital Bochum, Ruhr University Bochum, Bochum, Germany; 6 Second Department of Neurology, National and Kapodistrian University of Athens, Athens, Greece; 7 Department of Neurology, University of Essen-Duisburg, Duisburg, Germany; 8 Senior Professor of Neurology, University of Heidelberg, Heidelberg, Germany

**Keywords:** Ischemic stroke, TIA, Hemorrhagic stroke, Administrative data, Health care structure, Health service research

## Abstract

**Background:**

Comprehensive administrative data on TIA and stroke cases and treatment modalities are fundamental for improving structural conditions and adjusting future strategies of stroke care.

**Methods:**

The nationwide administrative database (German federal statistical office) was used to extract all adult inpatient TIA and stroke cases and corresponding procedural codes for the period 2011–2017. Numbers were specified according to age, sex, stroke unit (SU) and critical care treatment (ICU), early transfer, and in-hospital mortality.

**Findings:**

Inpatient adult TIA/stroke cases increased from annually 102,406 / 250,199 (2011) to 106,245 / 264,208 (2017). 84% of strokes were ischemic (AIS) also having the highest relative increase most likely due to more accurate coding within the time period, 68.2% of AIS were treated on SUs. 78% of hemorrhagic strokes were intracerebral hematomas (ICH; rather than subarachnoid hemorrhages [SAH]). Hemorrhagic strokes were increasingly treated on SUs (32.6% [2011], 37.8% [2017]). 68.8% of SAH were treated on ICUs (ICH:36.3%, AIS:10.3%). Early transfer in AIS increased (2.0 to 3.1%). Hemorrhagic strokes were associated with higher in-hospital mortality (SAH:19.6%, ICH:28.2%, AIS:7.3%).

**Interpretation:**

The absolute increase of strokes presumably reflects the aging society and more awareness for cerebrovascular disease. The relative increase of AIS may be attributable to an increased neurological expertise. The increasing amount of early transfers in AIS reflects new specialized treatment options. Our findings reflect the need for structural adjustments in inpatient stroke care.

## Key messages

1. The numbers of treated adult TIA and strokes cases in German hospitals has further increased in the observed time period 2011–2017. The proportion of hemorrhagic to ischemic stroke has not changed.
2. The rate of treatment on specialized wards like strokes units (SU) and intensive care units (ICU) has further increased for both ischemic and hemorrhagic stroke.
3. Hemorrhagic strokes are more frequently treated on ICUs, whereas cerebral infarctions and TIAs are more often treated on SUs. Hemorrhagic strokes are associated with higher in-hospital mortality.
4. For AIS, early transfer, e.g., to specialized neuro-vascular centers providing mechanical thrombectomy has increased in the time period.
5. The analysis of timely evolution of administrative data is important for future adjustments of infrastructure for inpatient stroke care.

## Introduction

The Global Burden of Disease Study recently provided data on global, regional and country-specific epidemiological data of stroke [1–4]. Like in many other epidemiological analyses, insight in stroke epidemiology mostly relies on population-based, regionally limited, observational cohort or hospital-based registries, all of which bare specific constraints [5–7]. Due to the necessity to code both diagnoses and treatment procedures for reimbursement, the German DRG registry provides accurate and comprehensive data not only on all inpatient ischemic stroke / TIA cases and treatment modalities in German hospitals [8]. Analysis of all hospitalized stroke cases can provide new insights into evolving trends of both ischemic and hemorrhagic stroke subtypes in everyday practice. Therefore, data from the German federal statistical office of all adult stroke patients hospitalized from 2011 to 2017 were used to identify frequencies of all inpatient stroke subtypes as well as treatment modalities on specialized wards. In addition, age- and sex-related differences, early transfer rates and in-hospital mortality rates were evaluated.

## Methods

Analyses were based upon the latest German Diagnosis-Related Groups (G-DRG) data provided by the German federal statistical office (DRG-statistic, www.destatis.de) for the years 2011 to 2017. All in-patient stroke cases are encoded according to ICD-10-GM^1^ and relevant operating and procedure keys (OPS-301 codes) issued by the German Institute of Medical Documentation and Information (DIMDI). Here, the following ICD main diagnosis codes were considered: G45.0-G45.99 (transient ischemic attack, TIA); I60.0-I60.9 (subarachnoid hemorrhage, SAH); I61.0-I61.9 (intracerebral hemorrhage, ICH); I63.0-I63.9 (cerebral infarction, AIS); I64 (stroke, not specified as hemorrhage or infarction). All case numbers were aggregated at the level of the 3-digit ICD codes. The age-standardized rates were calculated using the standard population of Germany based on the census of 2011 (Federal Statistical Office: Statistics on Natural Population Movement) [9]. In addition, the following OPS codes in combination with each considered main diagnosis were analyzed for all stroke subtypes^2^: 8–980 (basic intensive-care treatment); 8-98f (complex intensive-care treatment; from 2013 onwards); 8–981.0 (stroke unit treatment for more than 24 h and less than 72 h); 8–981.1 (stroke unit treatment for more than 72 h); 8-98b.*0/*1 (other acute stroke treatment without / with tele-consultation). For some analyses, OPS 8–980 and 8-98f were combined, as were the 3 subtypes of Stroke Unit care. Both first-ever and recurrent stroke cases were included, because the coding system cannot differentiate between them. Likewise, recurrent cerebrovascular events during hospital stay could not be analysed because these events are not coded consistently as a separate secondary diagnosis. Patients being transferred between hospitals during one treatment episode, (discharge key 06; transfer to another hospital), were censored accordingly in order to avoid any possible double/multiple coding. In addition, we assessed the number of acute stroke patients being transferred from one hospital to the other in the hyperacute phase for specific therapies such as mechanical thrombectomy, neurosurgical operations or intensive care treatment (so-called “hourly cases”). In-hospital mortality was assessed using discharge key 07 (death during hospital stay). For TIA and stroke subtypes, mean age with standard deviation, sex, and in-hospital mortality rate are provided. Only adult patients were considered.^3^ The maps of the regional frequency of ICH and AIS in Germany are based on the 413 administrative districts and independent cities in 2017. The age standardized rates are calculated for each district / city.

The pre-specified primary hypotheses were as follows:

1. The number of TIA and stroke cases (SAH, ICH, AIS, and non-specified stroke) treated in German hospitals has increased from 2011 to 2017.
2. The proportion of AIS vs. hemorrhagic strokes (SAH, ICH) has remained constant in the observed time period.
3. Hemorrhagic strokes are more frequently treated on intensive care units (ICU), whereas cerebral infarctions and TIAs are more often treated on strokes units (SU).
4. Hemorrhagic strokes are associated with higher in-hospital mortality.
5. For AIS, early transfer to specialized neuro-vascular centers providing mechanical thrombectomy has increased.

For descriptive analyses, results are reported as absolute numbers and percentages. Age bracketing of results (20–44, 45–59, 60–69, 70–79, 80–89, ≥90 years of age) was determined before analysis. Statistical comparison of groups was performed with Chi-square-test and Yates´ correction. Taking into account multiple testing and the very high number of cases, only *p*-values of < 0.001 were considered statistically significant. To evaluate potential differences between hemorrhagic and ischemic stroke, we estimated the odds ratios (ORs) with the corresponding 95% confidence intervals (CIs) for each outcome of interest. Cumulative estimates were pooled under the random effects model. Both within and between group differences in all analyses were assessed with the Cochran’s test for heterogeneity. All analyses were performed with IBM SPSS Version 25 and the Stata Statistical Software Release 13 for Windows (College Station, TX, StataCorp LP).

## Results

**Fig. 1:**
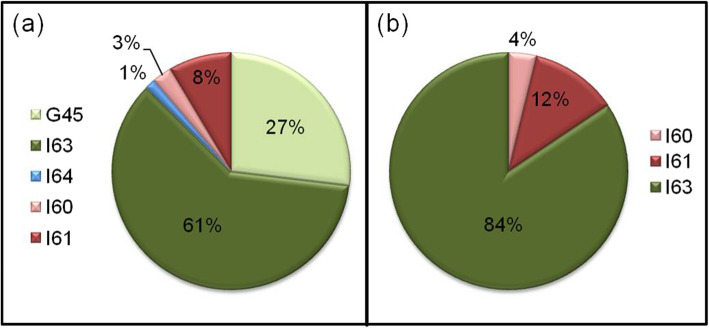
Distribution of inpatient TIA and stroke cases by diagnostic sub-types in Germany between 2011 and 2017, *n* = 2.853.370. **a**) considering the spectrum of diagnosis typically being treated on SUs and ICUs including TIA (ICD G45, I60, I61, I63, and I64). **b**) only considering hemorrhagic and ischemic stroke diagnoses (ICD I60, I61, and I63). G45 = transient ischemic attack, TIA; I63 = acute ischemic stroke, AIS; I64 = stroke not specified; I60 = subarachnoid hemorrhage, SAH; I61 = intracerebral hematoma, ICH

From 2011 to 2017, 2,544,850 cases of TIA (740,720) and stroke (1,804,130) of any kind (SAH, ICH, AIS, and non-specified strokes) have been treated in German hospitals. The annual number of TIA and stroke cases increased by 3.7 and 5.6% (TIA^4^: from 102,406 to 106,245; Stroke: from 250,199 to 264,208). AIS accounted for about 84% of all stroke cases. 78% of all hemorrhagic strokes were ICH. Detailed treatment numbers for different subtypes of stroke according to ICD codes are displayed in Table 1. Characteristic numbers comparing AIS (ICD I63) and ICH (ICD I61) cases are given in Table 2. The distribution of hospitalized patients with TIA and stroke subtypes is shown in Fig. 1. As most of epidemiological studies do not include patients with TIA, we present two illustrations with/without TIA cases.

**Table 1:** Administrative data of patients treated and coded as different DRG-driven stroke subtypes in German hospitals in 2011, 2014, and 2017. Proportions of specific treatment types driven by respective OPS codes without patients not treated in specific units

ICD code	G45 (TIA)			I60 (SAH)			I61 (ICH)			I63 (AIS)			I64 (n.s.)		
OPS code	ICU	SU	oSU	ICU	SU	oSU	ICU	SU	oSU	ICU	SU	oSU	ICU	SU	oSU
2011, *n*= f./ 00.000	102.406 **128**			7.427 **9**			25.841 **32**			209.766 **261**			7.165 **9**		
age, m ± SD female % early transf.% ih-mort.%	72.1 ± 4.9 53.9 1.9 0.4			59.1 ± 3.0 62,5 24.8 20.6			72.1 ± 5.1 48.6 13.5 28.8			74.1 ± 5.2 50.7 2.0 7.7			78.6 ± 6.0 58.9 17.1 15.9		
OPS %	3.0	43.5	5.0	64.1	12.5	0.4	35.5	35.5	2.8	9.3	55.2	5.4	5.8	5.6	6.8
2014, n= f. / 100.000	108.458 **134**			7.560 **9**			26.761 **33**			218.144 **269**			4.045 **5**		
age, m ± SD female % early transf.% ih-mort.%	72.0 ± 4.9 52.9 2.1 0.3			60.1 ± 3.1 60.5 22.1 18.9			72.8 ± 5.2 48.0 12.8 28.0			74.0 ± 5.1 49.3 2.3 7.0			78.9 ± 6.1 56.8 25.6 16.3		
OPS %	3.1	51.2	8.2	64.9	15.7	0.6	36.6	38.7	3.7	9.4	60.7	8.2	7.0	6.4	9.9
2017, n= f. / 100.000	106.245 **128**			7.547 **9**			27.036 **33**			227.542 **275**			2.083 **3**		
age, m ± SD female % early transf.% ih-mort.%	72.0 ± 4.9 52.2 2.2 0.3			61.1 ± 3.2 61.1 19.9 19.4			73.1 ± 5.3 47.3 12.6 29.3			74.0 ± 5.1 47.8 3.1 7.2			78.2 ± 5.8 53.4 39.6 15.4		
OPS %	3.3	56.6	9.1	66.8	18.4	0.6	36.3	40.1	3.0	10.3	65.3	8.2	4.8	9.1	8.3

ICD, international classification of diseases; TIA, transient ischemic attack; SAH, subarachnoid hemorrhage; ICH, intracranial hemorrhage; AIS, acute ischemic stroke; n.s., not specified; OPS, operating and procedure keys; ICU, OPS codes 8–980/98f; SU, stroke unit treatment OPS codes 8–981.0/−1; oSU, other stroke unit treatment OPS codes 8-98b.*0/− 1; f., raw frequency / 100.000 inhabitants without early transfer; m, mean; SD, standard deviation; early trans., early transfer within hours; ih-mort., in-hospital mortality

**Table 2:** Treatment characteristics comparing patients treated in German hospitals for intracerebral hemorrhage (I61) and acute ischemic stroke (I63) between 2011 and 2017 (*n* = 1.721.447)

variable	ICH (%, 95%PI) (ICD I61)	AIS (%, 95%PI) (ICD I63)	OR (95%CI)
In-hospital mortality	**28.1% (6–47%)**	**7.3% (0–15%)**	**6.43 (5.98–6.92)**
Δ 2017 vs. 2011 Δ expected	+ 5.7% + 1.9%	−6.3% −7.0%	
Total ICU treatment	**36.2% (11–64%)**	**9.7% (6–14%)**	**4.91 (4.39–5.48)**
basic ICU treatment (OPS 8–980)	17.9% (0–39%)	5.3% (0–10%)	3.31 (2.89–3.79)
complex ICU treatment (OPS 8-98f)*	18.3% (3–50%)	4.4% (5–7%)	5.05 (4.49–5.68)
Total SU treatment	**41.6% (34–46%)**	**68.2% (56–81%)**	**0.30 (0.28–0.32)**
SU treatment < 72 h (OPS 8–981.0	9.9% (6–13%)	29.6% (26–34%)	0.25 (0.23–0.27)
SU treatment > 72 h (OPS 8–981.1	31.7% (23–37%)	38.6% (28–49%)	0.69 (0.67–0.72)

ICH, intracerebral hemorrhage; AIS, acute ischemic stroke; OR, odds ratio; Δ expected, relative difference between age-adjusted expected rate for 2017 with reference to 2011; * case numbers OPS 8-98f in 2013–2017, see “Methods” section

Table 3 displays age-adjusted inpatient rates per 100,000 inhabitants and year for every stroke subtype by sex. Women were more often hospitalized for TIA (52.2% vs. 47.8%) and SAH (61.1% vs. 38.9%), whereas men were more often hospitalized for AIS (52.2% vs. 47.8%) and ICH (52.7% vs. 47.3%). The percentage of hemorrhagic strokes treated on ICUs remained stable (41.9% [2011] and 43.0% [2017). Patients with ICH had a 4.9 times higher odds ratio (95%CI 4.4–5.5) to be treated on an ICU compared with AIS, which was more pronounced for younger age (see Additional file 1: Figure S1). SAH patients were more often admitted to ICUs than ICH patients (67% vs. 36% [2017]). Hemorrhagic strokes were increasingly treated on stroke units (SAH: 12.8% [2011] and 19.0% [2017]; ICH: 38.3% [2011] and 43.1% [2017]). This effect was pronounced for increasing age. Otherwise, patients with AIS and TIA were predominantly treated on SUs (73.5 and 65.6% [2017]) and only 10.3% of AIS were treated on ICUs in 2017 (see also Additional file 2: Figure S2).

**Table 3:** Age adjusted inpatient rates / 100.000 inhabitants of patients treated and coded as different DRG-driven stroke subtypes in German hospitals in 2011, 2014, and 2017, according to sex

ICD	G45 (TIA)			I60 (SAH)			I61 (ICH)			I63 (AIS)			I64 (other)		
	t	m	w	t	m	w	t	m	w	t	m	w	t	m	w
2011	127	119	134	9	7	11	32	34	31	259	261	257	9	7	10
2014	128	122	134	9	7	11	32	33	30	256	263	250	5	4	5
2017	121	115	126	9	7	11	31	32	29	256	268	244	2	2	3

ICD, international classification of diseases; TIA, transient ischemic attack; SAH, subarachnoid hemorrhage; ICH, intracranial hemorrhage; AIS, acute ischemic stroke; t, total; m, male; f, female

The proportion of AIS treated on “other stroke units” increased up to 2014 and stayed constant since then, accounting for 8.2% of all cases with AIS in 2017 (in 2011: without/with tele-consultation: 4.6/0.9%; in 2017: without/with tele-consultation: 5.2/3.0%). Early acute transfer to another hospital for further treatment increased only for AIS from 2.0 [2011] to 3.1% [2017], while for hemorrhagic stroke there was a decrease of transfer rates (24.8 to 19.9% for SAH, 13.5 to 12.6% in ICH). In-hospital mortality was highest for hemorrhagic stroke (SAH 19.6% / ICH 29.3% [2017]) compared to AIS (7.2%) and TIA (0.3%). The risk for in-hospital mortality was 6.4 fold (95%CI 5.98–9.92) higher for hemorrhagic stroke (see Additional file 3: Figure S3), especially in younger patients. In ICH, in-hospital mortality increased from 28.8% (2011) to 29.3% (2017), in AIS, in-hospital mortality decreased from 7.7% (2011) to 7.2% (2017).

## Discussion

**Fig. 2:**
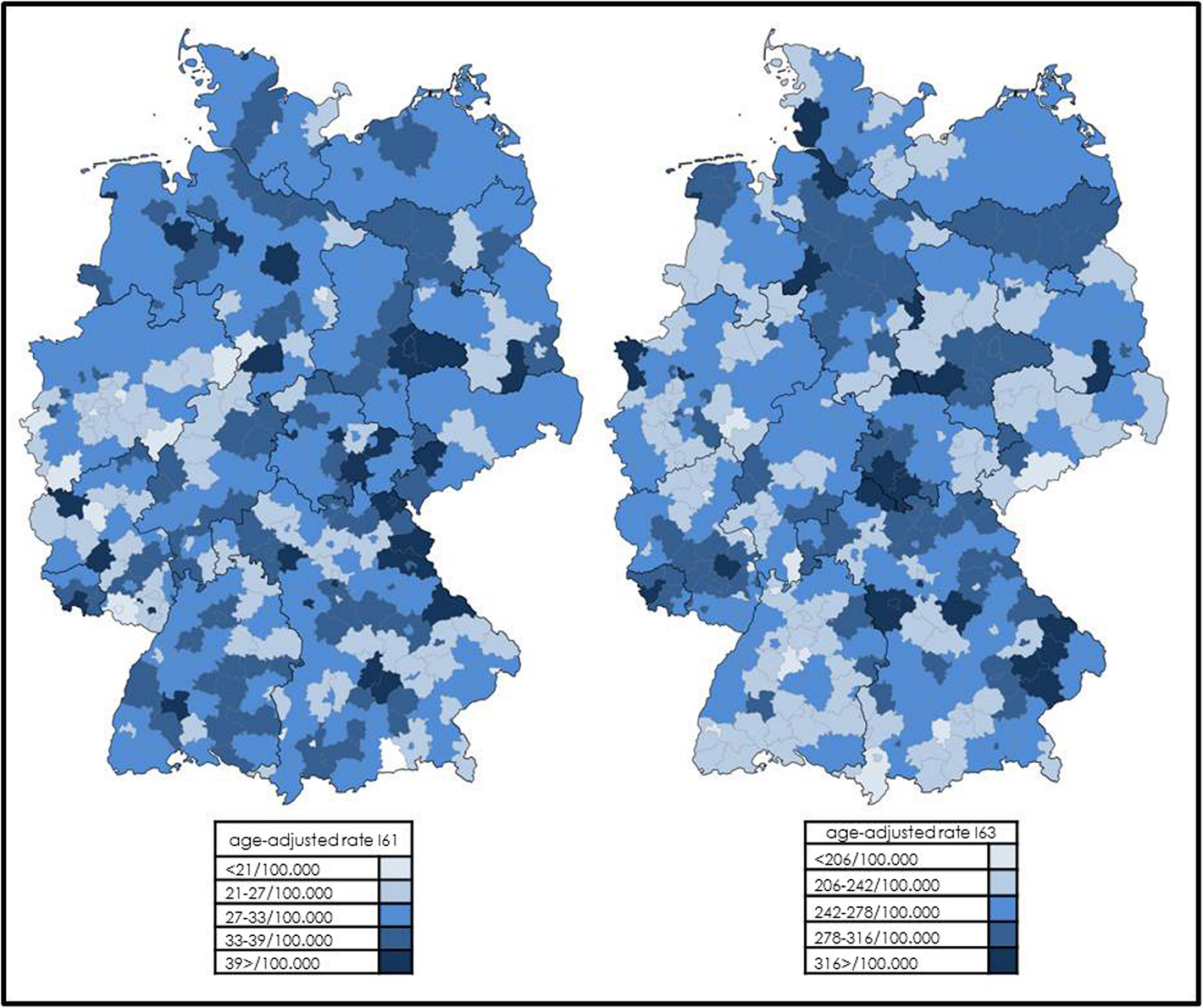
Distribution of age-adjusted inpatient rates for ICH (I61) and AIS (I63) in Germany in 2017 according to 413* administrative districts and cities according to patients´ places of residence. I61: median 31/100.000, mean 31/100.000, sd 5.9, range 11–54/100.000. I63: median 259/100.000, mean 262/100.000, sd 36.0, range 141–450/100.000. * 12 districts of Berlin are condensed to one for graphic reasons

Our analyses refer to all hospitalized adult TIA and stroke cases in Germany from 2011 to 2017, in total representing 2.55 million cases. Due to the specific characteristics of the German coding system, our data represent a robust image of the inpatient incidence and treatment reality of TIA/stroke in Germany. Our results confirm previously published data and trends [18], but also cast some genuine insights on structural conditions. The age-adjusted inpatient rate for SAH of 9.0 per 100,000 inhabitants in 2017, with more women being affected, is in line with previous data [11]. Since 10–15% of SAH die before hospital admission [17], a slightly higher incidence has to be assumed. The age-adjusted rates for hospitalized ICH and AIS were 31 and 256 per 100,000 inhabitants in 2017. Assuming a 10 year recurrence rate of up to 39% [12], the rate of first-ever ischemic or hemorrhagic stroke has to be reduced accordingly in our cohort. The Global Burden of Stroke study reported a worldwide age-adjusted annual first-ever stroke incidence rate of 258 in 2010 [1], varying between 217 and 281 depending mainly on income status of the respective country. However, treatment data such as ours is difficult to compare with epidemiological data. In addition, the epidemiological term “stroke” used by the Global Burden of Disease Study Group excluded TIAs but included ICH and SAH [10]. Considering coded TIA cases is controversial due to numerous differential diagnoses. Béjot et al. reported on a varying age-adjusted incidence rate for stroke and TIA in Europe between of 95 and 290/100,000 and 28 and 59/100,000, respectively, with higher incidence in eastern countries and lower incidence in southern countries for the early twenty-first century [5]. We also found a regional heterogeneity in the inpatient rates within Germany and have illustrated this in Fig.2 by graphic demonstration for ICH and AIS referring to all 413 administrative districts and cities of the country [8]. Our data cannot reveal, however, whether these differences are real differences in incidence, e.g., due to socio-economic variety or if this is solely bias. In the mentioned review of population-based studies it is also reported that age-adjusted incidence rates in men are 1.2 to 2 times higher than in women, possibly attributable to a higher prevalence of traditional vascular risk factors in men [5]. In our analysis we also found this difference for AIS and ICH, which even increased between 2011 and 2017. As for the proportions of different stroke subtypes, reviews describe wide variations for AIS from 55 to 90% of all cases, ICH from 10 to 25% and SAH from 0.5 to 5% [5]. In-hospital mortality slightly decreased in AIS patients during the observed time period, which can be explained by improved treatment strategies for AIS (e.g. higher use of thrombolysis, mechanical thrombectomy and hemicraniectomy). In contrast, in-hospital mortality slightly increased in ICH patients from 2011 to 2017, which might be attributed to an increasing use of oral anticoagulation in the general population [19].

Most of the review data refer to population-based, regionally limited, observational cohorts or hospital-based registries. Limitations of these study types have been discussed as focusing on urban areas, covering relatively small populations thus not reflecting the “true” composition of the population. Furthermore, the above mentioned studies have not analyzed the dispersion of treatment modalities such as specialized stroke unit (SU) or intensive care (ICU), which is a beneficial factor for patients´ outcome. Given high hospitalization rates in Germany, the German DRG statistic has proved as a useful tool to generate valid data on both (diagnoses-related) inpatient rates as well as the distribution of (OPS code-related) treatment modalities such as i.v. thrombolysis, mechanical thrombectomy, or ICU- and SU-care [8, 18], because correct and complete coding of both DRG- and OPS-codes is a prerequisite for reimbursement. While comprehensiveness of data is facilitated hereby, false economic incentives can be triggered. The German DRG-system is closely guarded, though, by a specialized medical service of the public health insurance system to avoid false coding. We therefore believe that most of the limitations discussed for administrative coding data (16) do not apply to the German system. Another inherent limitation, however, is the limitation to cases coded as such. Considering early transfers between hospitals in the first few hours, cases are considered as a transfer once they are coded as inpatient cases in the primary hospital (so called “hourly cases”). If they are not coded as an “inpatient” but only as an “outpatient” case, they will not appear as a transfer in the dataset, but as a regularly inpatient case of the secondary hospital. Regulatory rules provide the first mentioned proceeding even though it is unknown if all hospitals claim this proceeding. Therefore, transfer rates might be underestimated. However, we believe that due to reimbursement reasons every hospital has an inherent motivation to code these patients as “inpatient” cases. It can further be speculated if decreasing rates of early transfer rates as displayed may reflect improved allocation strategies of regional alliances between hospitals and emergency services, resulting in fewer necessary secondary transfers. Furthermore, our analysis is restricted to hospitalized cases only and cannot differentiate between first-ever and recurrent stroke. However, it is known that administrative data may result in an underestimation of disease incidence [13]. Further limitations include, e.g., the lack of data on stroke severity, vascular risk factors, medications, or functional outcome. Also, ethnicity, neuroimaging status, and other individual items cannot be accounted for due to the strict anonymization of the data set.

Our analyses provide genuine insights in the treatment and care reality in German hospitals. This is due to the fact that treatments on specialized wards like SU and ICU are separately reimbursed once coded in the system. Even though special requirements are to be fulfilled, it can be assumed that reimbursement is an effective incentive to provide any specialized treatment modality. We have formerly reported on treatment rates of systemic thrombolysis and mechanical thrombectomy in ischemic stroke and were able to show, that both rates have continuously risen in the past years to rates of 15.9 and 5.8% in 2017 [8, 14]. Treatment rates on specialized units also increased over the observed time period. The demonstrated numbers illustrate that SU treatment is not limited to ischemic strokes alone and that the care reality reflects the according recommendations of the professional societies. In Germany, currently there are more than 325 SUs certified by the German stroke society with a constant rise over the last 20 years [15]. One reason was the nationwide implementation of systemic thrombolysis and mechanical thrombectomy during this time period [8, 14]. AIS patients therefore are increasingly transferred to another hospital in the acute phase of treatment, mostly for interventional treatment. In order to guarantee for a comprehensive ability for interventional therapy over the country it is fundamental to provide a close net of SUs also in rural areas that can select patients qualifying for interventional therapy. This is partly met by networks encompassing “other stroke units” (i.e., not run by a neurological department), where neurological and neuro-radiological expertise are established by tele-medicine. We therefore hypothesized, that the amount of treatments on “other stroke units” should have increased in the observed time period. This has only been the case until 2014. We believe that this putative stagnation illustrates, that hospitals with formerly “other stroke units” have established a neurological department in the meantime.

## Conclusions

Our data provide important information on the development and trends in stroke care in Germany. These insights are essential for future adjustments of infrastructure for inpatient stroke care. As recommended by the professional societies, increasing numbers of stroke patients are treated on specialized units like certified stroke units or ICUs. In AIS, the increasing portfolio of therapeutic options like systemic thrombolysis and mechanical thrombectomy illustrates the need to improve access to specialized care. Therefore, our data mandate future efforts to further increase the proportion of stroke patients admitted to specialized units.

## Supplementary information

**Additional file 1: Figure S1.** Odds ratio (OR) of admission to intensive care unit (ICU) of patients treated for intracerebral hemorrhage (ICH) compared to patients treated for acute ischemic stroke (AIS) in German hospitals during the years 2011–2017, stratified by age group. Right favors ICH to ICU, left favors AIS to ICU. Dotted line represents overall OR including all ages and years.

**Additional file 2: Figure S2.** Stroke Unit (SU) admission rates of patients treated for acute ischemic stroke (AIS) in German hospitals during the years 2011–2017, stratified by age group.

**Additional file 3: Figure S3.** Odds ratio (OR) of all-cause in-hospital mortality of patients treated for intracerebral hemorrhage (ICH) compared to patients treated for acute ischemic stroke (AIS) hospitalized in German hospitals during the years 2011–2017, stratified by age group. Right favors mortality in ICH, left favors mortality in AIS. Dotted line represents overall OR including all ages and years.

## Data Availability

Availability of data is outlined in the methods section: DRG-statistic, Federal Statistical Office, www.destatis.de.

## Footnotes

1 International Statistical Classification of Diseases and Related Health Problems 10th revision, German modification

2 The German coding system and the ICD version remained unchanged during the analyzed time period except for implementation of the OPS code 8-98f for elaborated intensive care in 2013. Until 2012, all patients treated on an intensive care unit were coded with OPS 8–980.

3 Only few (< 600 per year) children and adolescents < 20 years were hospitalized for TIA or stroke between 2011 and 2017 and therefore excluded from statistical analysis.

4 Including G45.3 (amaurosis fugax) and G45.4 (transient global amnesia); G45.3 accounted for about 3.000 cases and G45.4 for about 7.000 cases.
