# Supplementary figures and images for: Inpatient TIA and stroke care in adult patients in Germany - retrospective analysis of nationwide administrative data sets of 2011 to 2017
Source: Neurol Res Pract. 2019 Dec 1;1:39. doi: 10.1186/s42466-019-0044-y (PMC7650112; doi:10.1186/s42466-019-0044-y)

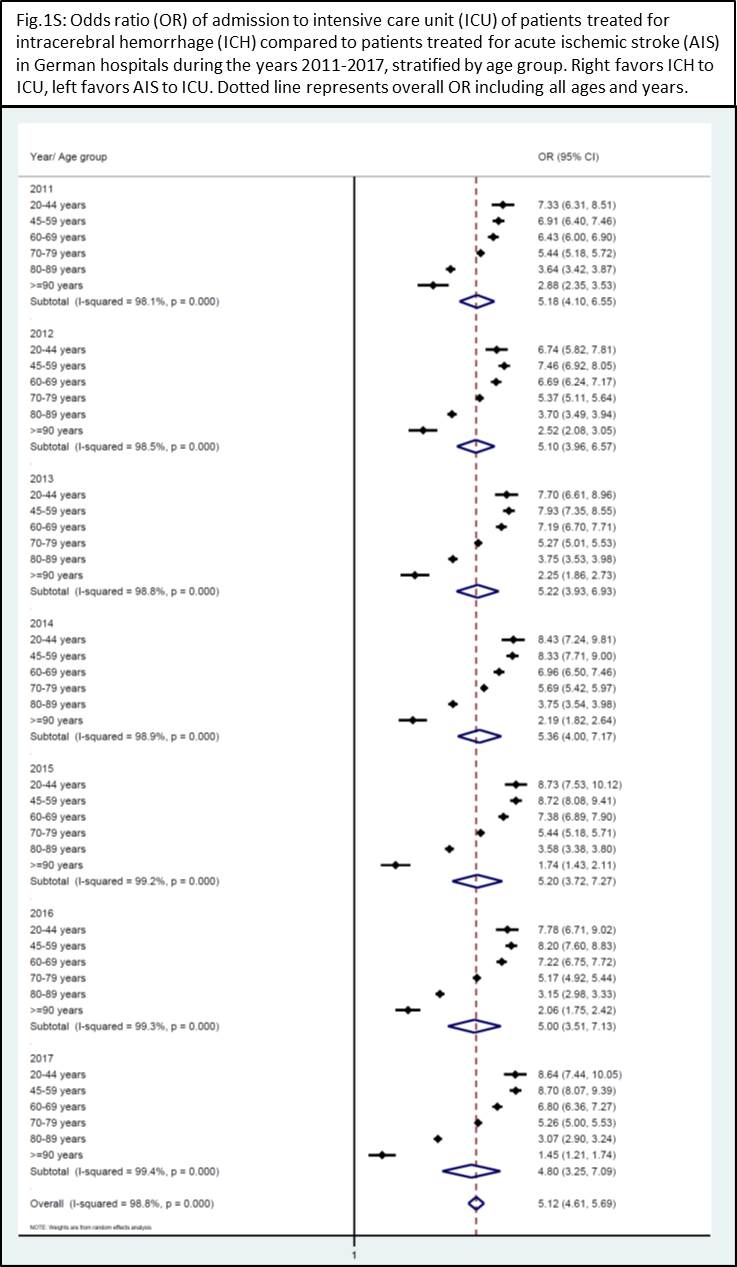

Supplement: Supplementary file 1 — Additional file 1: Figure S1. Odds ratio (OR) of admission to intensive care unit (ICU) of patients treated for intracerebral hemorrhage (ICH) compared to patients treated for acute ischemic stroke (AIS) in German hospitals during the years 2011–2017, stratified by age group. Right favors ICH to ICU, left favors AIS to ICU. Dotted line represents overall OR including all ages and years. [file 42466_2019_44_MOESM1_ESM.jpg]

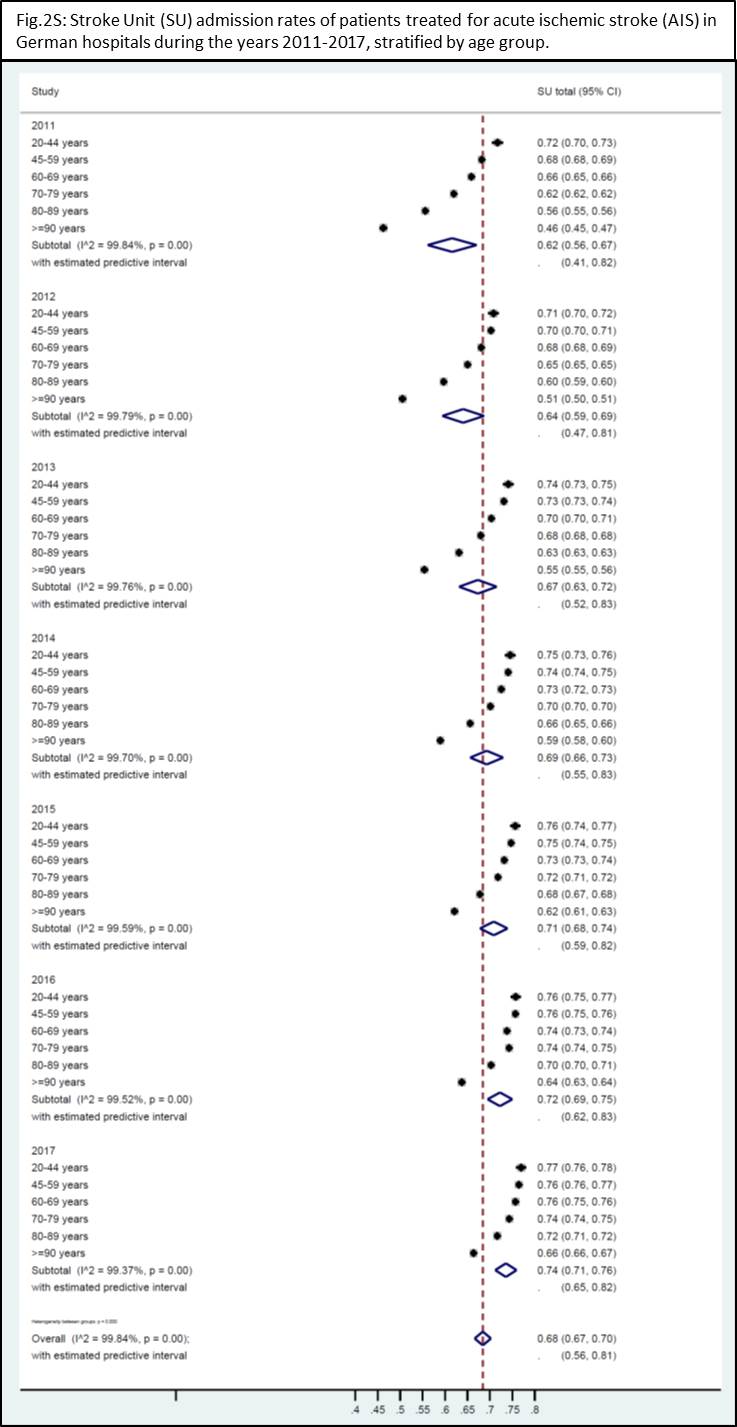

Supplement: Supplementary file 2 — Additional file 2: Figure S2. Stroke Unit (SU) admission rates of patients treated for acute ischemic stroke (AIS) in German hospitals during the years 2011–2017, stratified by age group. [file 42466_2019_44_MOESM2_ESM.jpg]

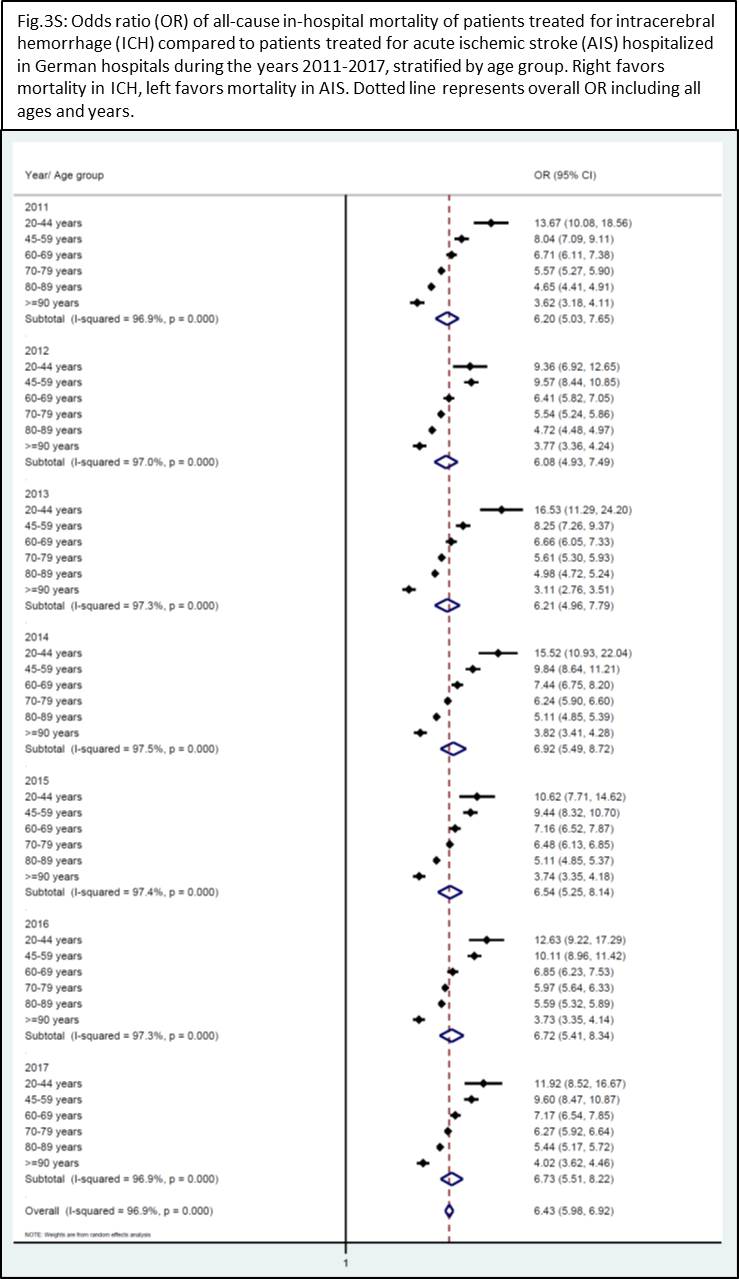

Supplement: Supplementary file 3 — Additional file 3: Figure S3. Odds ratio (OR) of all-cause in-hospital mortality of patients treated for intracerebral hemorrhage (ICH) compared to patients treated for acute ischemic stroke (AIS) hospitalized in German hospitals during the years 2011–2017, stratified by age group. Right favors mortality in ICH, left favors mortality in AIS. Dotted line represents overall OR including all ages and years. [file 42466_2019_44_MOESM3_ESM.jpg]
